# Supplementary material for: Seminoma and Embryonal Carcinoma Footprints Identified by Analysis of Integrated Genome-Wide Epigenetic and Expression Profiles of Germ Cell Cancer Cell Lines
Source: PLoS One. 2014 Jun 2;9(6):e98330. doi: 10.1371/journal.pone.0098330 (PMC4041891; doi:10.1371/journal.pone.0098330)
Supplement: File S1 — ZIP file containing DMRforPairs output for significant regions. Please start from the html files. (ZIP) [file pone.0098330.s008.zip › figures/16872.pdf]

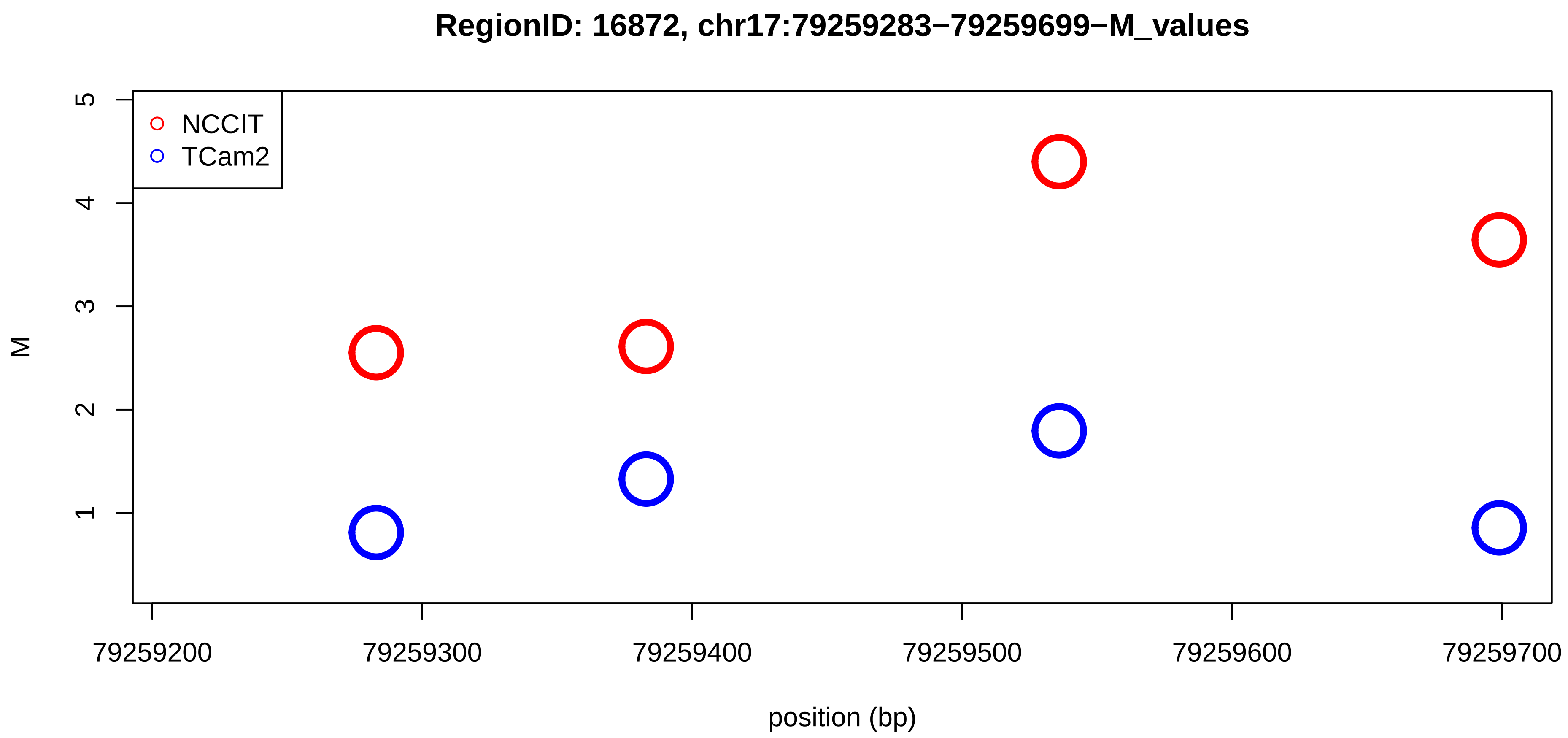
RegionID: 16872, chr17:79259283−79259699−M_values
NCCIT
TCam2
M
1
2
3
4
5
79259200
79259300
79259400
79259500
79259600
79259700
position (bp)

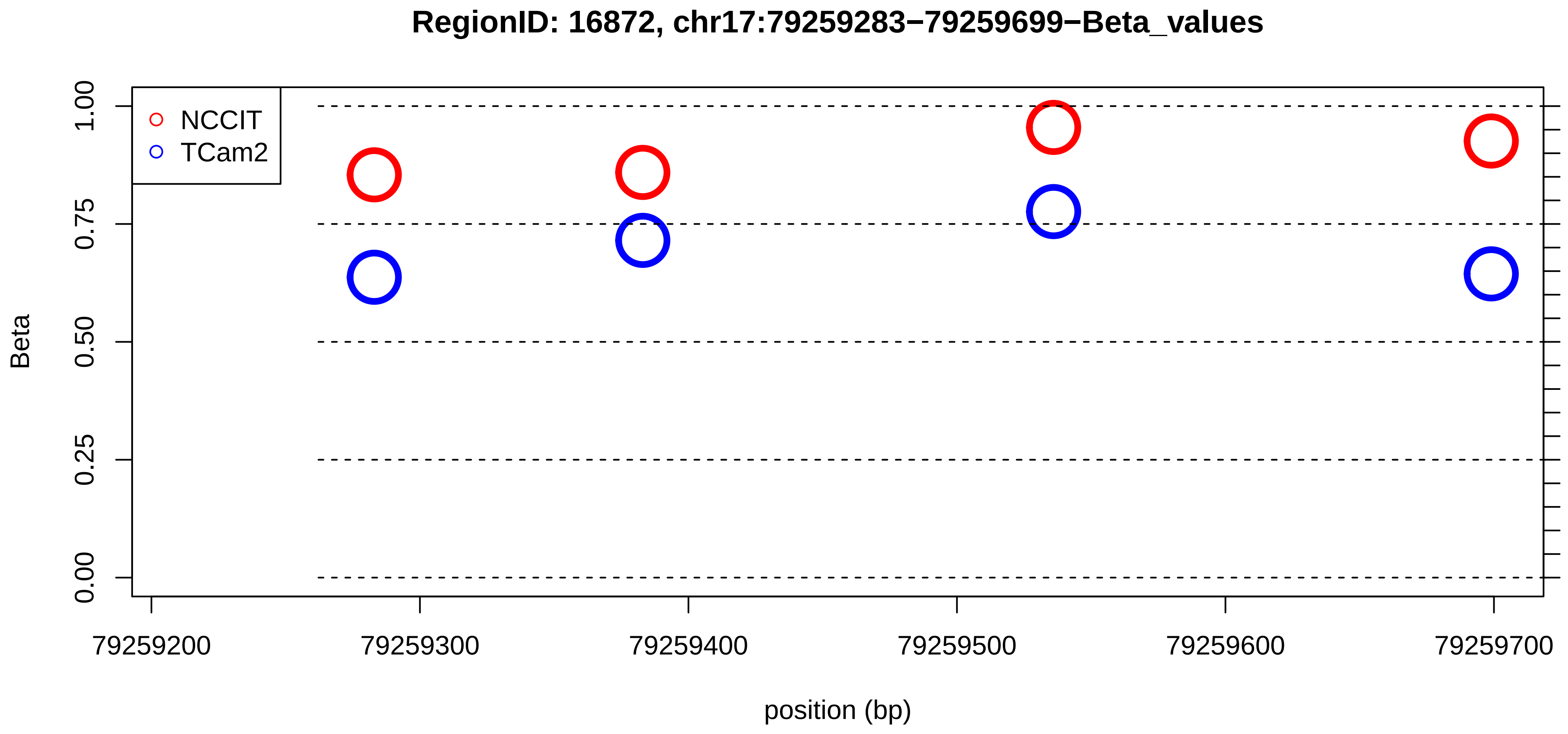
RegionID: 16872, chr17:79259283−79259699−Beta_values
NCCIT
TCam2
Beta
0.00
0.25
0.50
0.75
1.00
79259200
79259300
79259400
79259500
79259600
79259700
position (bp)
